# Anomalous Left Main Coronary Artery: Case Series of Different Courses and Literature Review

**DOI:** 10.1155/2013/380952

**Published:** 2013-12-22

**Authors:** Adam T. Marler, Jamil A. Malik, Ahmad M. Slim

**Affiliations:** Cardiology Service, San Antonio Military Medical Center, Fort Sam Houston, TX 78234, USA

## Abstract

*Background*. Congenital anomalies of the coronary arteries are a cause of sudden cardiac death. Of the known anatomic variants, anomalous origination of a coronary artery from an opposite sinus of Valsalva (ACAOS) remains the main focus of debate. *Case Series*. We present three cases, all presenting to our facility within one week's time, of patients with newly discovered anomalous origination of the left coronary artery from the right sinus of Valsalva (L-ACAOS). All patients underwent cardiac computed tomography for evaluation of coronary anatomy along with other forms of functional testing. Despite the high risk nature of two of the anomalies, the patients are being treated medically without recurrence of symptoms. *Summary*. After review of the literature, we have found that the risk of sudden cardiac death in patients with congenital coronary anomalies, even among variants considered the highest risk, may be overestimated. In addition, the exact prevalence of coronary anomalies in the general population is currently underestimated. A national coronary artery anomaly registry based on cardiac computed tomography and invasive coronary angiography data would be helpful in advancing our understanding of these cardiac peculiarities. The true prevalence of congenital coronary anomalies and overall risk of sudden cardiac death in this population are not well known. Surgical intervention remains the mainstay of therapy in certain patients though recent investigations into the pathophysiology of these abnormalities have shown that the risk of surgery may outweigh the minimal reduction in risk of sudden cardiac death.

## 1. Background

Coronary artery anomalies remain an important cause of debilitating cardiac symptoms and in some instances sudden cardiac death. Anomalies are most often classified into abnormalities of origin, distribution, and association with fistulae when present. The precise prevalence of coronary artery anomalies is not well defined. A study completed by Yamanaka and Hobbs in 1990 found the overall incidence of coronary artery anomalies in more than 120,000 patients undergoing coronary angiography to be 1.3% [1]. In a retrospective review of the Department of Defense data from 1977 to 2001, nontraumatic sudden death occurred in 126 military recruits out of over 6 million cases reviewed. Of these patients, the cause of death was identified as a coronary artery anomaly in 39 patients with 21 of those having an anomalous coronary artery origin. All 21 of these patients were found to have an anomalous origination of the left coronary artery from the right sinus of Valsalva (L-ACAOS) with an interarterial course between the pulmonary artery and aorta [2, 3].

Anomalous origin of the left coronary artery from the right sinus of Valsalva may be further separated into four separate subtypes. (1) The left main coronary artery courses between the aorta and the pulmonary artery. (2) The left main coronary artery tracks anteriorly over the right ventricular outflow tract. (3) The left main coronary artery takes an intramyocardial course before resurfacing at the proximal portion of the interventricular groove. (4) The left main coronary artery passes posteriorly around the aortic root [4]. Of these, the first anomalous configuration is classically considered the most dangerous placing patients at the highest risk of sudden cardiac death [5]. In this paper, we share examples of three of the four subtypes of anomalous origin of the left coronary artery from the right sinus of Valsalva.

## 2. Case Series

**Figure 1:**
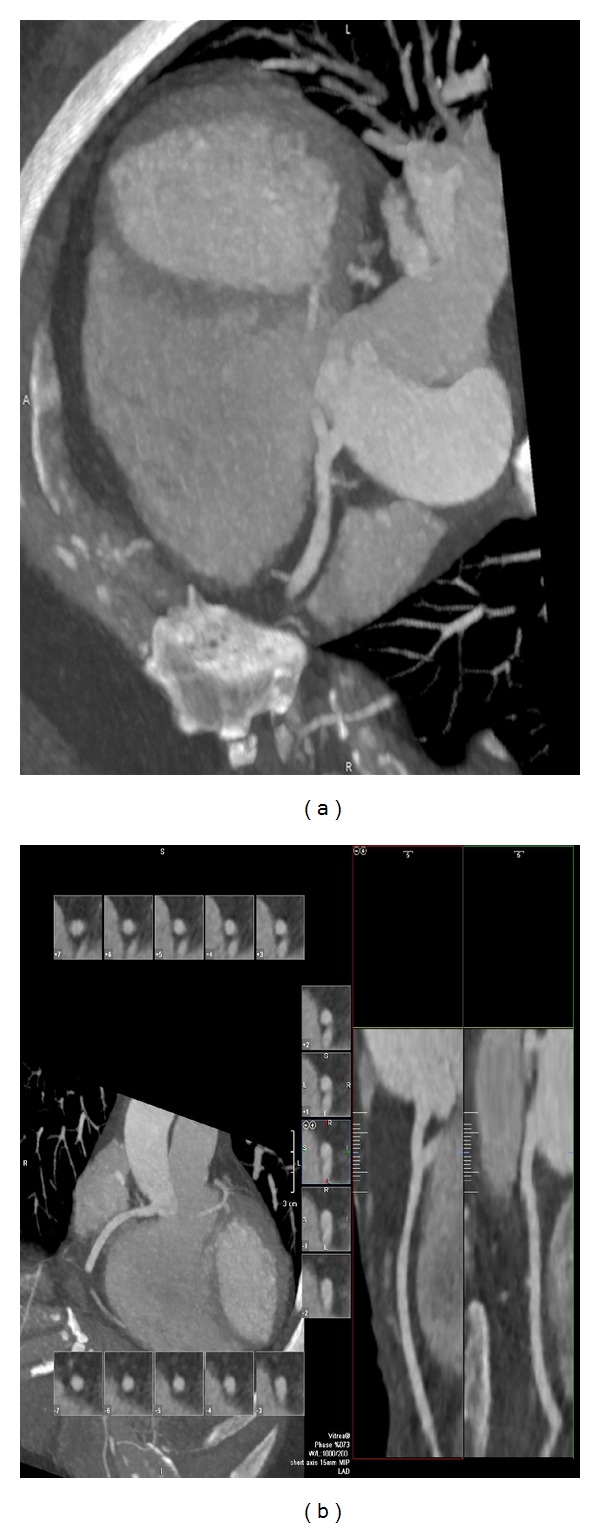
Cardiac computed tomography showing anomalous left main off of the right sinus of Valsalva and subsequent intraseptal course.

**Figure 2:**
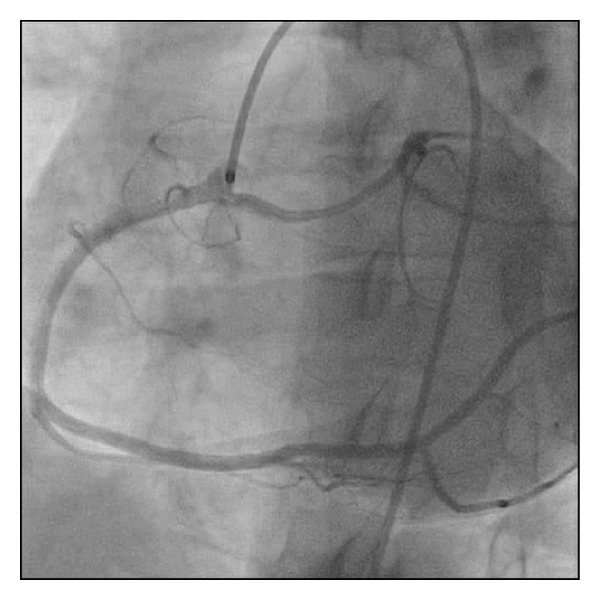
Left anterior oblique view of left main coronary artery with an anomalous origin off the right sinus of Valsalva with intraseptal course.

*Case  1*. A 42-year-old female with hypertension and hyperlipidemia was admitted to our facility for atypical chest pain. There was no reported history of syncope. Serial cardiac biomarkers were normal and the patient underwent coronary CT angiogram for assessment of coronary anatomy. Cardiac CT revealed no significant coronary artery disease but did show a left main coronary artery with an anomalous origin off of the right sinus of Valsalva with subsequent intraseptal course (Figure 1). Invasive coronary angiography (Figure 2) was completed with normal fractional flow reserve measurements in the proximal left anterior descending with adenosine infusion. The patient is currently doing well without further symptoms on optimal medical therapy alone.

**Figure 3:**
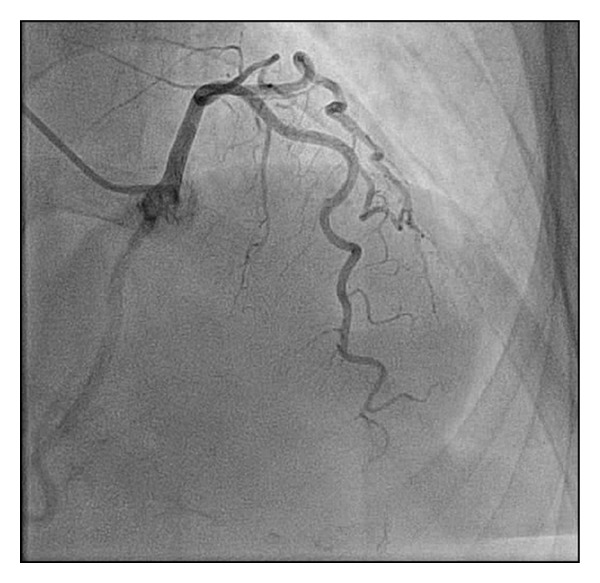
Right anterior oblique-cranial view of left main coronary artery with an anomalous origin off the right sinus of Valsalva with interarterial course.

**Figure 4:**
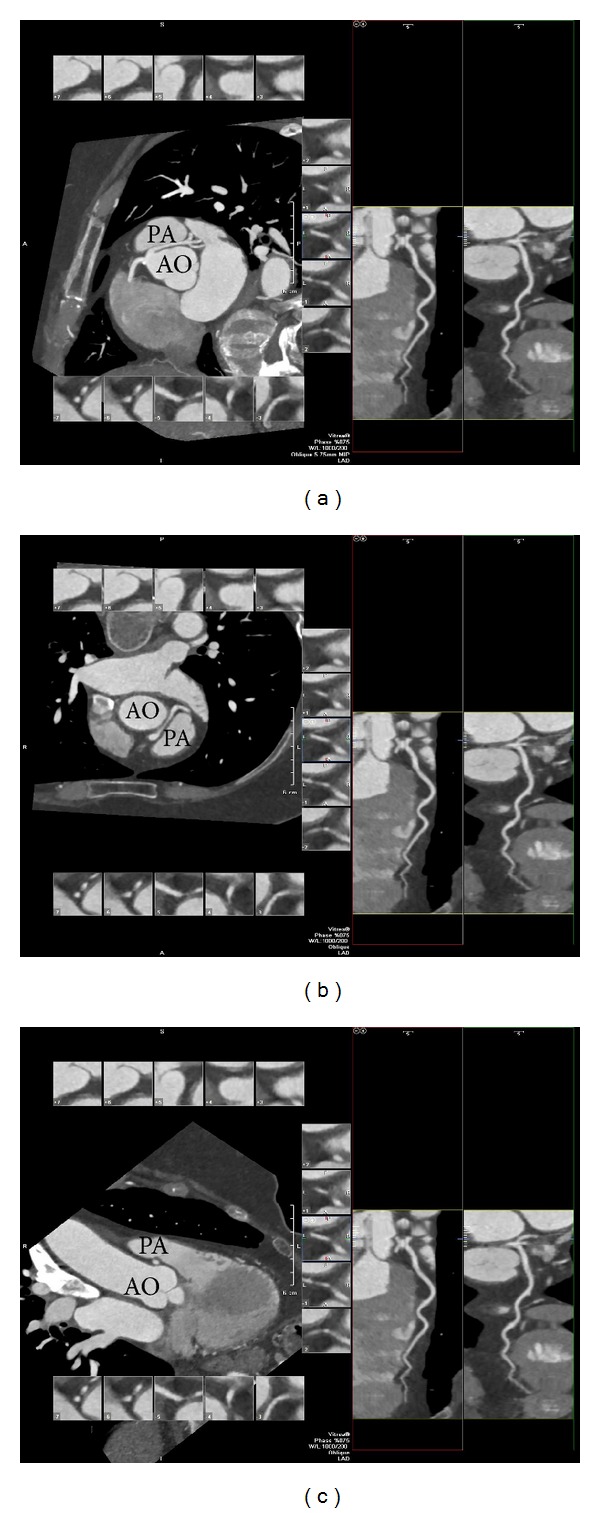
Cardiac computed tomography images of anomalous left main coronary artery off of the right sinus of Valsalva with a course between the pulmonary artery (PA) and aorta (AO).

**Figure 5:**
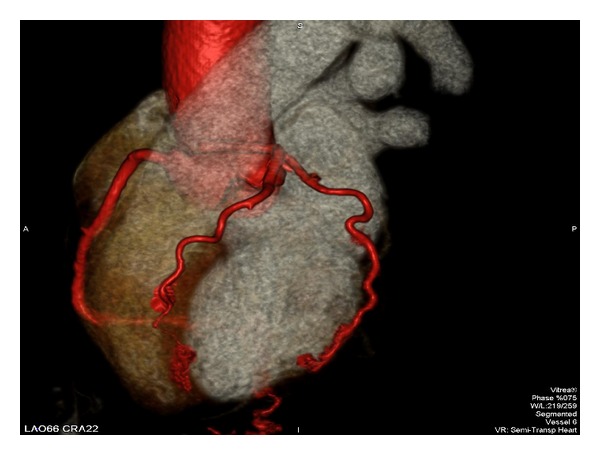
Three dimensional reconstruction exhibiting the interarterial course of the left main coronary artery as presented in Case 2.

*Case  2*. A 72-year-old female with hypertension and hyperlipidemia was admitted to the chest pain unit for evaluation of atypical chest pain. There was no reported history of lightheadedness, dizziness, or syncope. The patient underwent a stress echocardiography with no ischemic changes on electrocardiogram and no angina with evidence of mid anteroseptal wall hypokinesis at peak exercise. The patient underwent invasive coronary angiography (Figure 3) showing no significant coronary artery disease and a left main coronary artery with an anomalous takeoff from the right coronary cusp. The myocardial course of the left main was unable to be definitively determined by invasive coronary angiography and cardiac CT was completed. Cardiac CT showed the left main to have an anomalous takeoff from the right coronary cusp with a course between the great vessels (Figures 4 and 5). The patient is currently asymptomatic on maximal medical therapy.

**Figure 6:**
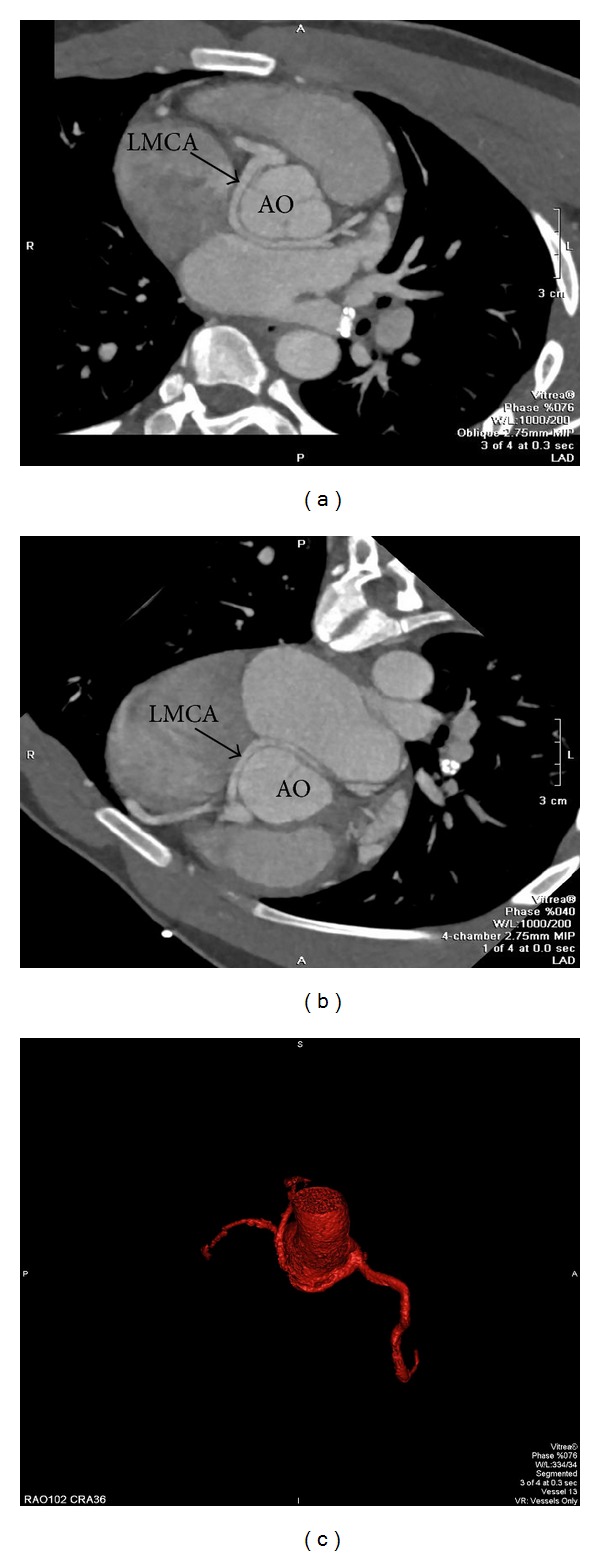
Cardiac computed tomography and three dimensional reconstruction showing an anomalous left main coronary artery (LMCA) off of the right sinus of Valsalva assuming a posterior course around the aorta (AO).

*Case  3*. This is a 21-year-old case of a male with exercise limiting chest pain and no reported history of syncope. The patient reported intermittent chest pain since the age of 16 which had become worse since the beginning of the military training. The patient underwent coronary CT angiogram showing a left main coronary artery with an anomalous takeoff from the right coronary cusp. The left main coronary artery (LMCA) was seen to take a posterior course behind the ascending aorta before bifurcating into the left anterior descending and left circumflex arteries (Figure 6). Exercise stress testing was completed with myocardial perfusion imaging during which the patient exercised for 14 minutes and 35 seconds (14.4 METS) with no ischemic EKG changes and no angina. Myocardial perfusion images showed no evidence for ischemia, normal left ventricular ejection fraction, and normal wall motion. The patient was started on no additional medical therapy and continues to experience some symptoms with heavy exertion.

## 3. Discussion

Coronary artery anomalies represent a life-threatening form of congenital cardiac pathology. The underlying cause of sudden cardiac death in patients with congenital coronary abnormalities is the source of some debate with multiple competing theories. Death may result from contortion of the vessel's slit-like, tangential origin during exercise leading to ischemia and resultant arrhythmia. Another theory purports that compression of the anomalous coronary occurs with dilation of the aorta and pulmonary artery during exercise. It is also possible that frequent episodes of myocardial ischemia lead to myocardial fibrosis and potential nidus for a deadly arrhythmia [3, 6]. Finally, Anjelini and associates evaluated three patients with L-ACAOS by intravascular ultrasound both at rest and after pharmacologic challenge with saline/atropine/dobutamine (“SAD” test). The group discovered the severity of ischemia to be related to degree of coronary intussusception, level of intramural hypoplasia, and lateral compression of the vessel by expansion of the aorta [2, 5, 6]. The true cause of death is likely an aggregate form of the currently proposed theories.

In addition to contention over the true mechanism of death related to coronary artery anomalies, the true prevalence of disease within the general population has remained a mystery. Congenital coronary anomalies are likely under-recognized as completing an anatomic assessment in a very large portion of the population would seem unfeasible. As mentioned above, the angiographic study completed by Yamanaka and Hobbs found the overall incidence of coronary artery anomalies in a population of more than 120,000 patients to be 1.3%. In this same study, “benign” coronary anomalies were the most frequently discovered with an incidence of 1.07%. L-ACAOS was found in only 22 patients comprising a fairly small portion of abnormalities considered potentially serious [1]. If these same statistics were theoretically applied to the 6.3 million military recruits reviewed by Eckart et al., the overall number of the expected coronary artery anomalies would be 81,900. In the same study, the expected number of military recruits with L-ACAOS would be 1,071. In total, the review found only 21 deaths attributable to an anomalous coronary artery origin, all of which were the L-ACAOS variant. This suggests that the prevalence of congenital coronary anomalies in the general population is much higher than previously predicted. Also, it is very likely that the overall risk of sudden cardiac death in patients with congenital coronary anomalies is much less than previously reported [3, 6]. In fact, this has been suggested by a group from the New York University School of Medicine in reviews published in 2005 as well as in 2012 [6, 7].

All three patients presented in this case series were found to have a different anatomic configuration of the L-ACAOS variant. Cases included one with an interarterial course, one with an intramural course, and one with a posterior course. Two of the three patients have remained symptom free since the initial presentation. None of the patients underwent surgical correction of their unique coronary anomaly as the risk of surgery outweighed the potential benefit of correction based on completed functional studies. None of the patients were evaluated by intravascular ultrasound as completed by Angelini's group in 2006 [2]. Of interest is the fact that the patient with the reportedly most benign variant, LMCA with a posterior course, developed the most severe symptoms likely related to his high level of activity as a military member. In contrast, the patient with an interarterial course, considered to be the most insidious variant, developed symptoms in the eighth decade of life potentially due to prolonged hypertension and associated changes in aortic morphology.

## 4. Conclusion

In conclusion, congenital coronary artery anomalies remain as a cause of sudden cardiac death though it appears that the risk is lower than previously thought. The true prevalence of coronary artery anomalies in the general population is unknown. In addition, the absolute risk of sudden cardiac death is difficult to predict and hinges upon multiple variables. Treatment of coronary artery anomalies is controversial and dependent on the discovered anatomy. Surgery is the mainstay of treatment though beta blockers and calcium channel blockers have been utilized to lessen ischemic symptoms. In the current case series, we present three patients of three different generations with three different variants of left coronary artery from the right sinus of Valsalva. Two of the three patients are currently being treated medically and continue to remain symptom free. As imaging with cardiac computed tomography continues, maintenance of a congenital coronary anomaly registry would help to broaden our medical knowledge and improve diagnostic and therapeutic modalities for future generations.

## References

[1] Yamanaka O, Hobbs RE (1990). Coronary artery anomalies in 126,595 patients undergoing coronary arteriography. *Catheterization and Cardiovascular Diagnosis*.

[2] Angelini P, Walmsley RP, Libreros A, Ott DA (2006). Symptomatic anomalous origination of the left coronary artery from the opposite sinus of valsalva: clinical presentations, diagnosis, and surgical repair. *Texas Heart Institute Journal*.

[3] Eckart RE, Scoville SL, Campbell CL (2004). Sudden death in young adults: a 25-year review of autopsies in military recruits. *Annals of Internal Medicine*.

[4] Hauser M (2005). Congenital anomalies of the coronary arteries. *Heart*.

[5] Angelini P (2009). Anomalous origin of the left coronary artery from the opposite sinus of valsalva: typical and atypical features. *Texas Heart Institute Journal*.

[6] Peñalver JM, Mosca RS, Weitz D, Phoon CK (2012). Anomalous aortic origin of coronary arteries from the opposite sinus: a critical appraisal of risk. *BMC Cardiovascular Disorders*.

[7] Mirchandani S, Phoon CKL (2005). Management of anomalous coronary arteries from the contralateral sinus. *International Journal of Cardiology*.

